# The new dialysis method Mini-SLED is useful for dialyzing acute brain stroke patients

**DOI:** 10.1186/cc10977

**Published:** 2012-03-20

**Authors:** F Taki, Y Komatsu

**Affiliations:** 1 St Luke's International Hospital, Tokyo, Japan

## Introduction

Hemodialysis (HD) patients are known to be a high-risk population for brain stroke. On acute phase of stroke, standard HD treatment may increase cerebral damage by changing serum and tissue osmolarity. For low clearance dialysis, CRRT, PD or low Qb HD were used but there are some complications. To dialyze these patients more safely and simply, we modified a new dialysis method, Mini-SLED (sustained low-efficiency dialysis).

## Methods

We conducted a retrospective observational study from June 2006 to October 2011. Maintenance HD patients who onset acute brain stroke, including hemorrhage and ischemic infarction, were observed. We divided patients into four groups by dialysis modality and compared the clinical parameters. Determination of Mini-SLED was Qb 200 ml/minute, QD 100 to 200 ml/minute, duration for 2 to 3 hours.

## Results

Sixty-one patients were observed in this study. Mean age 72.5 years, 39 patients were male, 45 patients had diabetes. Major clinical parameters and outcomes are presented in Table 1. Patients treated with Mini-SLED have lower risk of rebleeding compared to low Qb HD or CRRT, and were more cost-effective than PD. Delivered Kt/V of Mini-SLED was 0.72 ± 0.23. Modality difference did not affect mortality.

**Table 1:** Dialysis Methods and clinical parameter

	CRRT	PD	Low Qb HD	Mini-SLED
	(*n *= 25)	(*n *= 3)	(*n *= 21)	(*n *= 12)
NIHSS (score)	30.8 ± 17.2	34.6 ± 16.4	31.7 ± 20.8	32 ± 19.8
Rebleed (*n*, %)	6, 24%	1, 33%	4, 19.0%	0, 0%
Mortality (*n*, %)	5, 20%	1, 33%	5, 23.8%	2, 16%
Kt/V (daily)	0.68 ± 0.32	0.25 ± 0.16	0.86 ± 0.23	0.72 ± 0.23
Cost ($/1 treat)	498 ± 30.2	92.4 ± 22.6	82.5 ± 12.5	86.7 ± 14.3

## Conclusion

Our Mini-SLED methods are effective and safe for dialyzing acute brain stroke patients.

